# Interpreting NADH fluorescence lifetime across timescales

**DOI:** 10.1038/s42003-026-11344-z

**Published:** 2026-11-12

**Authors:** Gianmarco Ferri, Francesco Cardarelli

**Affiliations:** 1 Fondazione Pisana per la Scienza ETS, San Giuliano Terme, Italy; 2 Laboratorio NEST, Scuola Normale Superiore, Pisa, Italy

**Keywords:** Biological fluorescence, Bioenergetics, Cellular imaging

## Abstract

NADH fluorescence lifetime imaging is a widely used, label-free way to visualize cell metabolism, typically read as a balance between glycolysis and oxidative respiration. This reading assumes that the effective NADH-binding landscape remains sufficiently stable during the experiment. This assumption is generally best protected during brief perturbations, but can fail in two ways: slowly, as prolonged treatments or differentiation reshape the identity and abundance of NADH-binding enzymes over hours to days; and rapidly, as metabolites can transiently alter their engagement with NADH even without changes in protein abundance. In this Perspective, we outline when each concern applies and offer practical guidance for experimental design and interpretation.

## Introduction

**Fig. 1:**
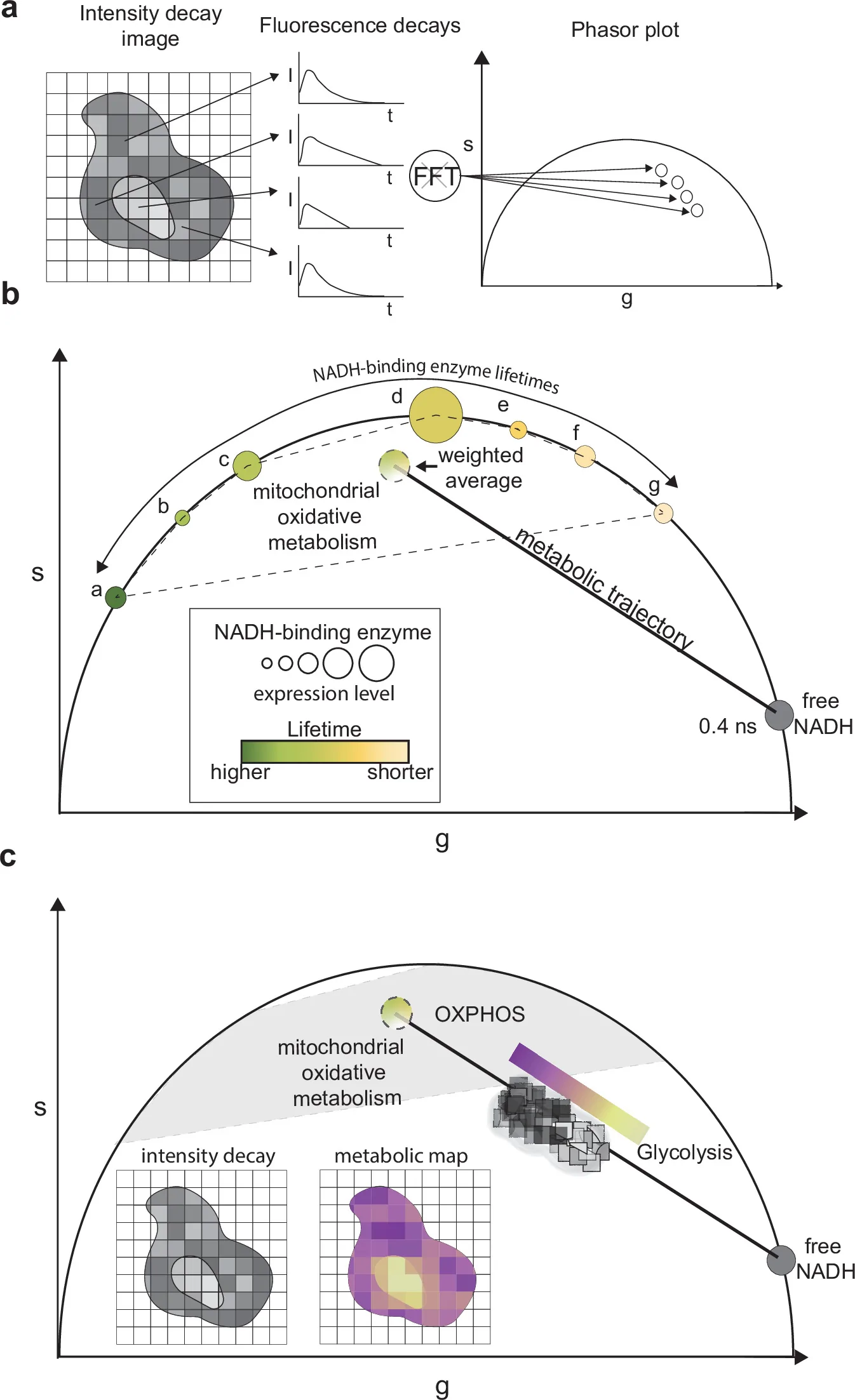
The phasor approach to NADH FLIM and the multi-enzymatic nature of the bound state. **a** Schematic overview of the phasor transform workflow, demonstrating the pixel-by-pixel transformation of fluorescence intensity decay profiles from a cellular image into discrete coordinates on the phasor plot. **b** Biophysical representation of the intracellular bound NADH state. Rather than defining a single, homogeneous biochemical state or a fixed universal coordinate on the universal semicircle, the bound component is a dynamic linear combination of distinct enzyme-bound lifetimes. The resulting bound reference point, labeled “weighted average” in the panel, represents the weighted average of these underlying oxidoreductase contributions, which varies inherently according to the specific proteomic context and engagement within individual cells. **c** The classical metabolic trajectory is established by connecting the free NADH lifetime component with the dynamic, multi-enzymatic bound NADH fraction. Shifts along this trajectory reflect changes in the relative fractions of free and bound coenzyme, enabling the geometric translation of raw lifetime data into a spatially resolved, color-coded metabolic map discriminating between glycolytic activity and oxidative phosphorylation. The trajectories and arrows shown are conceptual schematics illustrating the underlying mechanism, not universal or quantitatively precise representations of any single experiment.

The implementation of NADH fluorescence lifetime imaging (FLIM) provides a powerful, non-invasive means to monitor live-cell bioenergetics. This technique fundamentally relies on a well-established two-state framework derived from the biophysical properties of the coenzyme^1^: free NADH exhibits a short fluorescence lifetime (~0.4 ns), while enzyme-bound NADH exhibits longer lifetimes (~2–4 ns). Within this framework, the relative abundance of these two states provides a direct window into cellular bioenergetics: a shift toward the free component is classically interpreted as an increase in glycolytic activity, whereas a migration toward the bound component is viewed as a signature of oxidative phosphorylation^2^ (OXPHOS)—an assignment grounded empirically in experiments using pharmacological agents with well-defined mechanisms of action, as discussed below. Historically, metabolic imaging implemented this model through bi-exponential fitting of decay curves^3^, though since the landmark work of Stringari et al.^4^ the community has increasingly favored the phasor approach, a fit-free geometric method that transforms pixel-by-pixel fluorescence intensity decays into unique coordinates on a phasor plot (Fig. 1a).

This simplicity, however, rests on assumptions that deserve explicit consideration. At the molecular level, the bound lifetime component does not represent a single homogeneous state or a fixed, universal coordinate on the universal semicircle. Instead, it is a heterogeneous ensemble of enzyme-specific binding configurations (Fig. 1b): different oxidoreductases impose distinct conformational constraints on NADH, yielding a distribution of lifetimes whose weighted average reflects the underlying binding proteome^5^. In the phasor framework, this weighted average determines the actual position of the “intracellular bound NADH” pool (Fig. 1b). Crucially, this bound endpoint is inherently dynamic and context-dependent—shaped not only by which enzymes are present, but also by the local metabolic milieu in which they operate. Because the precise abundance and real-time engagement of specific enzymes cannot be easily measured a priori or known for individual cells, this point represents a complex linear combination rather than a rigid, uniform baseline. Representing it as a single static reference is an operational simplification; in reality, each cell possesses a unique enzymatic profile that shapes its specific bound NADH coordinate. This variability, however, is partly constrained by the strong contribution of mitochondrial Complex I to the NADH fluorescence signal. Binding to Complex I markedly enhances NADH fluorescence^6–8^, thereby helping to anchor the aggregate bound coordinate to a bioenergetically meaningful region of the phasor plot.

Connecting this anchored bound fraction with the free NADH component (0.4 ns) defines the metabolic trajectory, i.e., the geometric backbone of the phasor approach that translates raw lifetime data into an intuitive spatial representation (Fig. 1c). The biochemical and spatial basis for this anchoring is examined in the following section. The enduring appeal of this technique lies in its experimental accessibility: a single, label-free imaging session yields a quantitative and spatially resolved metabolic map through this elegant geometric framework.

In this Perspective, we examine the biophysical assumptions this interpretation rests on and ask when they hold. We argue that only one of them, that the population of NADH-binding enzymes remains stable, carries a genuine time-based guarantee, which is why interpretive risk scales primarily with the timescale of an experiment: acute perturbations are largely protected, prolonged treatments and differentiation are not, and even the acute regime carries residual, non-proteomic risks worth controlling for. Using a phasor-based simulation, we further show that even when the enzymatic landscape does change, the resulting drift in the bound-NADH coordinate can be quantified rather than simply treated as noise. We close with practical guidance for designing and reporting NADH FLIM experiments across these different regimes.

## The three pillars of NADH FLIM interpretation

Translating these analytical observations into definitive biological conclusions requires the framework to satisfy three foundational pillars—namely, a biochemical, a spatial, and a temporal assumption.

## The biochemical assumption

This assumption establishes a direct mapping between the physical state of the coenzyme and specific metabolic pathways. Within this classical framework, glycolysis is treated as the primary functional driver of the cytosolic free NADH pool, while mitochondrial Complex I is viewed as the definitive sink for the enzyme-bound fraction. Under standard conditions, changes in the free-to-bound ratio are thus assumed to mirror the balance between glycolytic activity and oxidative phosphorylation (OXPHOS). Critically, however, this interpretation rests on a fundamental biophysical distinction: FLIM does not directly monitor kinetic reaction rates (metabolic flux) but rather captures changes in the steady-state concentrations and saturation levels of the free and bound coenzyme pools. The canonical assignment of phasor displacements to glycolysis or OXPHOS is grounded in experiments using perturbations with well-characterized mechanisms of action, most notably respiratory chain inhibitors, such as rotenone, and glycolytic blockers, such as 2-deoxyglucose^9^. These studies established the empirical relationship between shifts in the free-to-bound NADH ratio and changes in cellular metabolic activity, providing the foundation for the metabolic interpretation of NADH FLIM. Although this dichotomous framework has found experimental validation, it conceals a straightforward biochemical simplification that is worth flagging to the reader. A non-negligible cytosolic-bound fraction exists alongside the mitochondrial one, contributed chiefly by lactate dehydrogenase and other cytosolic oxidoreductases; its relative weight is normally small under the mitochondrial dominance, but it need not stay small if cytosolic enzyme levels or engagement change selectively. The mitochondrial matrix itself is not a single homogeneous compartment, either: local differences in enzyme density and complex assembly across individual mitochondria and mitochondrial subregions mean that the aggregate bound coordinate is a population average over a heterogeneous population of binding sites, not a single well-defined value. This weighted average also depends on quantum yield, which need not be identical across bound states: enzymes whose non-radiative decay dominates emission—the common case for protein-bound NAD(P)H—have quantum yield approximately proportional to lifetime, a convention we adopt below for illustration rather than an experimentally established rule for every NADH-binding enzyme. This heterogeneity extends to the optical readout as well.

Additional contributions arise from NADPH, which is spectrally indistinguishable from NADH but metabolically distinct. Its contribution to the measured NAD(P)H autofluorescence is system- and compartment-dependent and cannot be assumed to be negligible, because shifts in the NADPH/NADH balance can substantially affect the observed lifetime distribution^10^. Similarly, further complexities stem from variations in total NAD(H) pool size, which can mimic redox alterations independently of flux^11,12^, as changes in absolute coenzyme abundance alter the saturation state of available intracellular binding sites. In complex tissue settings, further confounds include collagen, elastin, and flavins^13^, which introduce overlapping autofluorescence backgrounds that can artificially shift the composite lifetime coordinates on the phasor plot. Importantly, the commonly used biexponential model is a simplified projection of a richer set of binding states^14^, and this biophysical limitation is fundamentally independent of the downstream mathematical approach chosen for data analysis, whether fit-free geometric representations or numerical decay fitting. Thus, the challenge is inherently biological rather than algorithmic: any mathematical reduction of a multi-component, heterogeneous signal into a strict two-state framework risks conflating genuine flux changes with these compositional and optical confounds, regardless of the fitting method used.

## The spatial assumption

This assumption is closely tied to the cytosolic contribution just described and introduces an optical and topographical pre-filter to this metabolic mapping, asserting a strict compartmental bias. The cellular readout in standard FLIM experiments does not integrate the intracellular environment uniformly; instead, the large majority of detected autofluorescence photons originate strictly from the mitochondrial network. This spatial bias is driven both by the substantially higher concentration of the coenzyme within the mitochondrial matrix and by a physical fluorescence enhancement effect, where binding to Complex I markedly amplify the NADH quantum yield. Consequently, the optical readout is topographically pre-filtered to be predominantly sensitive to mitochondrial bioenergetics, effectively shielding the system from minor cytosolic variations under standard conditions. In practice, this mitochondrial-dominance argument is typically invoked and validated at the scale of the whole cell or the imaging field, often pooling many cells within a single frame—recent large-field optical platforms^15^ now extend this same population-level scale to entire cell colonies. Whether the same weighting holds equally within individual cells, or varies systematically across subcellular compartments, has not been addressed systematically. Direct validation at finer subcellular resolution—for instance across individual mitochondria, between matrix and intermembrane space, or in relation to the cytosolic contribution described above—remains comparatively limited and would be a natural target for dedicated compartment-resolved studies correlating optical readouts with independent biochemical measurements.

## The temporal assumption

This assumption requires that the underlying enzymatic binding landscape remains predominantly invariant over the timescale of the experiment, and in fact bundles two distinct requirements that are not automatically satisfied together: invariance in enzyme identity and abundance—which NADH-binding proteins are present, and how much of each—and invariance in binding configuration—the fraction of an already-present, unchanged enzyme pool that is actually engaged with NADH at a given moment, as opposed to free or occupied by a competing ligand. The former is protected, over short timescales, by the biological inertia of transcription and translation (see below); the latter is not automatically protected by that same inertia, since ligand binding and dissociation can occur on a timescale of seconds to minutes. A shift in the local concentration of a competing metabolite can therefore displace the bound-NADH reference point even when the enzymatic proteome itself has not changed at all; we return to this second axis below.

In more complex biological settings, lifetime changes may still report genuine metabolic alterations, but the specific pathways responsible for those changes cannot always be inferred from FLIM data alone. These complexities, however, do not invalidate the readout, but they do call for care in specifying which regime an experiment falls into. In typical acute perturbation experiments, the balance between glycolysis and OXPHOS indicators remains the dominant driver of lifetime variation: the biochemical and spatial assumptions hold to a good approximation, and the temporal assumption is protected, at least with respect to enzyme identity and abundance, by the biological inertia described below. Under these conditions, variations in lifetime can generally be assigned to steady-state shifts in the free-to-bound NADH balance, though not unconditionally, as the following sections discuss. Conversely, when the temporal assumption is violated in the stronger sense, as occurs when the enzymatic repertoire itself undergoes remodeling, the readout increasingly conflates these metabolic shifts with alterations in the binding context, and downstream mechanistic interpretation regarding pathway flux becomes correspondingly more ambiguous. This distinction between experimental regimes is a useful starting point, as discussed below, rather than a sharp unconditional boundary: of the three pillars, only the temporal one admits a genuine time-based guarantee, which is why the sections that follow are organized primarily around the acute-to-chronic axis.

## When the assumptions are best protected: the case for acute experiments

**Fig. 2:**
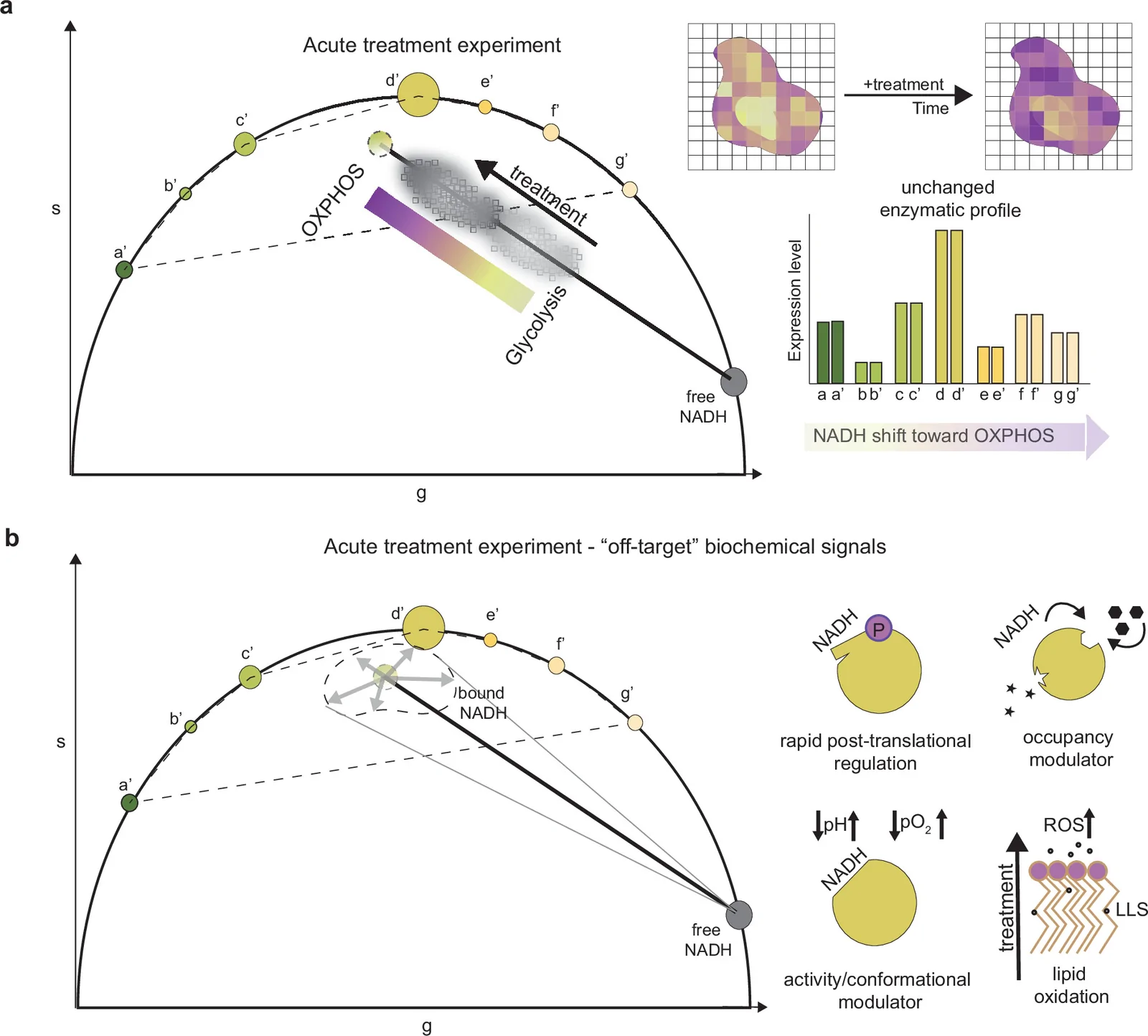
Distinguishing genuine flux changes from off-target biochemical signals in the acute regime. **a** In a classical acute perturbation, the enzymatic profile remains unchanged before and after treatment, so any displacement of the phasor cloud tracks the pre-existing metabolic trajectory between free NADH and the bound endpoint, faithfully reporting a shift in the balance between glycolysis and OXPHOS. **b** The same acute regime remains susceptible to several off-target biochemical signals that can displace the bound-NADH reference point without any change in enzyme abundance: an occupancy modulator (a competing metabolite redistributing an enzyme between binding states), an activity/conformational modulator (pH, oxygen tension), rapid post-translational regulation (e.g., phosphorylation), and lipid oxidation generating long-lifetime autofluorescent species (LLS) under oxidative stress. The trajectories and arrows shown are conceptual schematics illustrating the underlying mechanism, not universal or quantitatively precise representations of any single experiment.

The assumption of a stable enzymatic landscape is well-justified in a large and important class of experiments: those in which cells are imaged before and after an acute pharmacological perturbation or physiological stimulus. In mammalian cells, significant shifts in enzyme abundance typically require transcriptional and translational responses that unfold over hours or days; acute treatments act on existing enzymatic machinery within minutes, leaving that particular aspect of the protein landscape effectively invariant between measurements. This is a specific protection, not a general one: it addresses the first of the temporal assumption’s two requirements—identity and abundance—not the second, binding configuration. Nor does it touch the compositional limitation already noted for the biochemical assumption. Because the intracellular enzymatic profile can be considered unchanged, to a good approximation, between the pre- and post-treatment states (Fig. 2a), the coordinate of the multi-enzymatic bound NADH fraction on the phasor plot remains anchored close to its starting position. Any observed displacement of the phasor cloud tracks predominantly along the pre-existing, static metabolic trajectory (e.g., shifting toward OXPHOS), meaning that the recorded delta in lifetime reliably reports a shift in the balance between glycolytic and oxidative activity (Fig. 2a). The relevant timescale is not arbitrary, though it is best read as an approximate indicator of risk rather than a sharp cutoff: adaptive changes in enzyme abundance in mammalian cells typically become appreciable within roughly 6–24 h, depending on the stimulus and whether it engages transcriptional regulators of metabolic gene expression, such as HIF-1α, c-MYC, or PGC-1α^16,17^. Perturbations acting well below this threshold can therefore be interpreted with confidence within the stable-landscape assumption. While Niederschweiberer et al.^18^ formalized this logic through the concept of the “metabolic delta”— specifically the change in NADH lifetime following respiratory inhibition—the underlying paradigm of measuring rapid stimulus-induced changes is a common cornerstone of the field. This approach is implicitly utilized in numerous studies, such as those monitoring metabolic shifts in pancreatic β-cells^19,20^ where the immediate response to a treatment allows the cell to serve as its own internal control. By focusing on a short timescale, sources of variation that are insensitive to the acute perturbation, including protein composition and NADPH contributions, are effectively cancelled. In these settings, the two-state model is not merely a useful simplification; it is a well-grounded approximation where lifetime changes directly report the underlying free-to-bound redistribution, because the enzymatic context is held constant by biological inertia.

### Caveats even within the acute regime

This protection, however, only covers the first of the two requirements described above: it says nothing about binding configuration. A change in the local concentration of a metabolite that competes with NADH for the same enzyme can shift the occupancy of an already-present, unchanged enzyme pool within seconds, well inside the acute regime, displacing the bound-NADH reference point exactly as proteomic remodeling does over longer timescales, but for a different reason and on a different clock. The magnitude of this displacement is not the same for every enzyme, however: given the mitochondrial dominance established above, an occupancy shift at a minor cytosolic contributor such as LDH is expected to move the aggregate bound-NADH coordinate only modestly, whereas the same kind of shift acting on a mitochondrial oxidoreductase would be expected to carry disproportionately more weight. The acute regime is therefore best described as protected against confounding by proteomic drift, rather than immune to every source of drift in the bound endpoint. Figure 2b summarizes the resulting picture: alongside the occupancy shift just described, intracellular pH and oxygen tension act as activity/conformational modulators of the bound endpoint, and rapid post-translational regulation—phosphorylation and related covalent modifications—can alter enzyme-coenzyme binding on a comparably fast timescale, all without any change in protein abundance. None of these require new protein synthesis, so none are excluded by the biological-inertia argument; where a perturbation is expected to engage any of them, acute experiments should ideally include a mechanistically distinct control and report pH, oxygenation, or phosphorylation-state alongside the lifetime data. These signals are genuinely biochemical, not measurement artifacts—real consequences of the perturbation that happen to act on an axis different from the one being interrogated. We refer to them as off-target relative to the specific question of metabolic flux, following the same logic by which long-lifetime autofluorescent species generated by lipid oxidation under oxidative stress^21^, once treated as a phasor-analysis complication, have been reclassified as an informative oxidative-stress signal in their own right.

## Where the assumptions break down: prolonged treatment or long-term adaptation

Unlike the fast, off-target signals just described, which leave enzyme identity and abundance untouched, the temporal assumption’s first requirement—the one biological inertia does protect in the acute regime—eventually gives way once treatments or biological processes extend long enough for the enzymatic repertoire itself to change.

Mitochondrial dominance described above does not protect against interpretative ambiguity here; on the contrary, because Complex I anchors the aggregate signal so strongly, remodeling of the mitochondrial enzymatic landscape is reflected directly and maximally in the FLIM readout. Pharmacological treatments or biological adaptations extending over days inevitably engage transcriptional and translational pathways that alter the expression of both mitochondrial and cytosolic NADH-binding oxidoreductases; while cytosolic contributions to the total baseline signal may remain quantitatively minor, as noted above, structural remodeling of the mitochondrial compartment itself profoundly alters the aggregate bound lifetime profile.

**Fig. 3:**
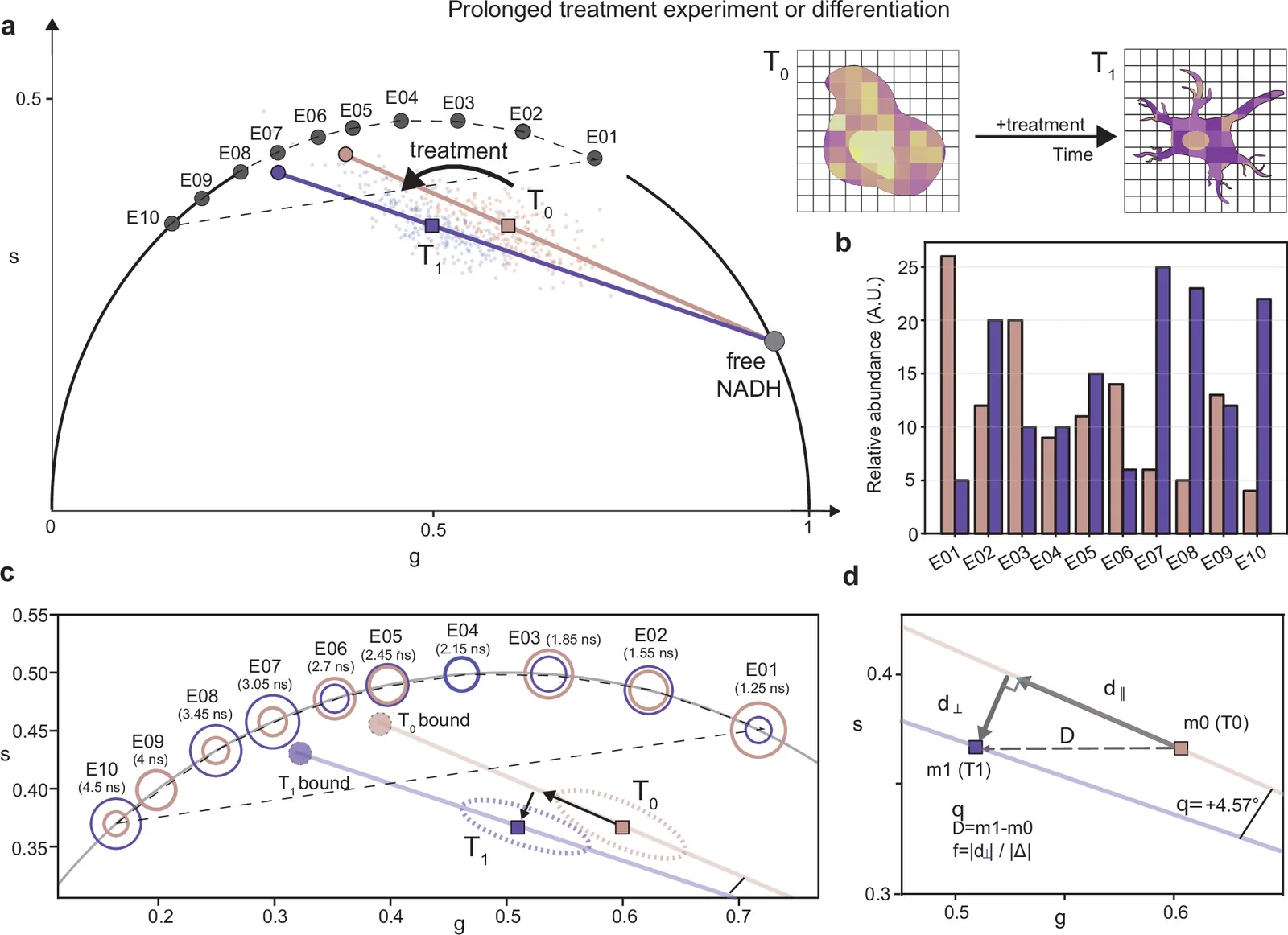
Proteomic remodeling displaces the bound-NADH endpoint over long timescales. **a** In prolonged treatment or differentiation experiments, the enzymatic profile itself changes between an initial (T0, pink) and final (T1, purple) time point, physically displacing the aggregate bound-NADH coordinate on the phasor plot and rotating the observed trajectory relative to its original position; a comparable rotation can also arise on much shorter timescales from ligand-driven shifts in binding-site occupancy alone, without any change in enzyme expression, as discussed in the main text. **b** Simulated relative abundance of ten representative NADH-binding enzymes (E01–E10) at T0 and T1, illustrating a representative pattern of proteomic remodeling used to generate the trajectories in **a**). **c** Detail of the phasor plot in (**a**), showing the fixed bound lifetime assigned to each reference enzyme (1.25–4.5 ns) and the resulting T0 and T1 measured centroids obtained from the simulated remodeling scenario, including pixel-level photon noise. **d** The displacement Δ between the T0 and T1 measured centroids is decomposed into a component parallel to the original trajectory (d∥, indistinguishable from a pure shift along that axis) and a component perpendicular to it (*d*⊥, a displacement incompatible with a pure along-axis shift; in this simulation, attributable to proteomic remodeling, the only mechanism modeled here), yielding a rotation angle *θ* and a fractional deviation *f* = |*d*⊥|/|Δ|, both quantifiable independently of the underlying flux change.

Two experimental scenarios are particularly relevant. The first is long-term differentiation^22–24^. During the transition from progenitor to specialized cell type, metabolic reprogramming is accompanied—and in part mediated—by coordinated changes in the expression of metabolic enzymes. The NADH-binding landscape at an early differentiation stage and at a late stage are not equivalent. A change in mean NADH lifetime measured between two differentiation timepoints therefore reflects a combination of altered metabolic flux and an altered ensemble of NADH-binding proteins. The second scenario is prolonged pharmacological treatment^25,26^. When cells are exposed to a drug for 24 h or more—a common experimental design in studies of metabolic drugs, chemotherapy, or nutritional interventions—the treatment may itself induce transcriptional responses that alter enzyme expression. In this case, the enzymatic landscapes before and after treatment are no longer equivalent. Interpreting a lifetime shift as a metabolic response to the drug conflates the direct effect on flux with any indirect effect mediated by changes in enzyme abundance. This critical conflation is visually apparent during prolonged treatments or differentiation processes (Fig. 3a). Over these extended timescales, transcriptional and translational remodeling rewires the underlying proteome, leading to a substantially altered enzymatic profile (Fig. 3b). This structural reorganization changes the weighted average of the bound lifetime components—potentially encompassing the de novo expression of previously absent enzyme isoforms or the complete downregulation of constitutively expressed ones—physically displacing the bound NADH reference point on the universal semicircle. Consequently, the phasor cloud does not merely slide along a fixed trajectory; instead, it transitions to an entirely new metabolic trajectory.

To illustrate this quantitatively, we constructed a reference population of ten NADH-binding enzymes with fixed, literature-derived bound lifetimes spanning the physiologically observed range (1.25–4.5 ns)^1,5,27^ (Fig. 3c) and simulated a representative remodeling scenario by resampling their relative abundance between an initial (T0) and a final (T1) time point (Fig. 3b). Because phasor coordinates add linearly, each scenario yields a well-defined centroid at both time points; even under a realistic pixel-level photon budget, the resulting T0 and T1 point clouds remain clearly separated on the phasor plot, with the T1 centroid departing from T0 along a direction that diverges from the original free-to-bound trajectory, rather than sliding along it (Fig. 3c). This departure is not an artifact of any particular choice of enzymes or abundances: it follows directly from the fact that a change in which enzymes dominate the bound population redefines what “bound” means on the phasor plot, independently of any change in flux. In a representative scenario, this remodeling produces a trajectory rotation of θ ≈ 4.6° at the free-NADH vertex, with roughly 40% of the total observed displacement lying perpendicular to the original trajectory rather than along it. Because live-cell FLIM measurements are themselves subject to photon-counting noise, we simulated pixel-level point clouds under a realistic photon budget (~10⁵ photons per condition, comparable to standard TCSPC acquisitions); despite this noise, the T0 and T1 clouds remain clearly separated on the phasor plot, with the two metabolic axes resolved at approximately 16 standard deviations—well above the noise floor for any acquisition delivering more than a few thousand photons per condition. We return below to how this departure can itself be measured. The distinction matters most when the goal is mechanistic: understanding whether a drug shifts cells toward OXPHOS is a different question from observing that it changes NADH lifetime. This limitation arises not only from experimental timescale, but more generally from comparisons across biological contexts with distinct enzymatic compositions. Direct experimental evidence in the context of long-term differentiation is provided by a recent longitudinal study of human neuronal differentiation^24^. In that work, stem cells were differentiated toward cortical neurons over 4 months, with NADH FLIM measurements performed monthly alongside quantitative proteomics targeting NADH-binding and metabolic enzymes. Mean NADH lifetime increased progressively across the differentiation time course, consistent with the expected shift toward oxidative metabolism in maturing neurons. However, quantitative proteomics revealed substantial concurrent remodeling of the NADH-binding enzymatic repertoire. The lifetime data, taken in isolation, do not resolve the question it was originally asked to answer.

## Beyond the breakdown: quantifying drift without resolving its cause

A more modest but still useful question is whether this departure can itself be turned into a quantitative readout, even when the specific remodeling scenario that produced it cannot be recovered. As Fig. 3 already illustrates, many different combinations of rising and falling enzyme abundances can produce a shift of comparable overall size; from lifetime data alone, there is generally no way to know which specific combination occurred, or how much the enzymatic landscape has changed in absolute terms. Rather than trying to reconstruct that combination, the displacement itself can be decomposed geometrically with respect to the original trajectory (Fig. 3d): a component d∥, running parallel to the original free-to-bound axis, is indistinguishable from a pure shift along that same axis, while a component *d*⊥, perpendicular to it, captures a displacement incompatible with a pure along-axis shift, attributable to remodeling of the enzymatic landscape in the simulation below, though in a real experiment it could equally reflect any of the off-target signals discussed above. Because phasor coordinates add linearly, both components are well defined for any observed displacement Δ between the initial and final measured centroids, yielding a rotation angle *θ* and a fractional deviation *f* = |*d*⊥|/|Δ| that together summarize how far, and in which direction, the interpretation has been pulled off the original trajectory. Both quantities depend on the modulation frequency used for acquisition: because the phasor transform maps a given lifetime to a different position on the universal semicircle depending on the laser’s modulation frequency^28^, the same underlying rotation can appear as a different *θ* or *f* when measured at a different frequency. *θ* and *f* should therefore be compared only across experiments run at the same or a documented modulation frequency, not treated as frequency-independent biological constants. Across the simulated remodeling scenarios, these two parameters remain stable and interpretable regardless of which specific combination of abundance changes produced a given displacement. An experiment that cannot recover which enzymes changed, or by how much, can therefore still report a quantitative estimate of how far its readout has departed from what a pure flux change would predict, without claiming to know why.

## Practical considerations for experimental design and reporting

The considerations above point to a small number of concrete choices that determine whether a given NADH FLIM experiment can support a mechanistic claim. Table 1 summarizes how interpretive risk scales with the experimental regime, from acute perturbations to chronic treatment and differentiation; Box 1 translates this into a practical checklist for experimental design, controls, and reporting.

**Table 1:** Experimental regimes, timescales, and interpretive risk for NADH FLIM

Regime	Typical examples	Main risk to the temporal assumption	Recommended safeguard
Acute (seconds–minutes)	Rotenone/2-deoxyglucose bolus, single-stimulus assays	Ligand- or metabolite-driven shift in binding configuration, even with enzyme abundance unchanged	Mechanistically distinct pharmacological control; paired pre/post design
Short (up to a few hours)	Single-dose drug exposure at a fixed time point	Generally low: enzyme abundance not yet remodeled	As above; report stimulus identity and exposure time
Intermediate (~6–24 h)	Sustained drug exposure, early differentiation	Onset of transcriptional/translational remodeling (e.g., HIF-1α, PGC-1α, c-MYC target genes)	Independent proteomic or transcriptomic check of enzymatic context
Long (>24–72 h and beyond)	Chronic treatment, full differentiation, chemotherapy response	Substantial proteomic remodeling expected; flux and enzymatic context become inseparable from lifetime data alone	Quantitative proteomics/transcriptomics strongly recommended before making any mechanistic claim

Box 1: Practical checklist for NADH FLIM experimental design

- Define the experimental regime first. Acute perturbations (minutes to a few hours) and comparisons across biological states (differentiation stages, chronic treatments, distinct cell types or tissues) rest on different assumptions and carry different risk.
- Separate observation from mechanism. Reporting that NADH lifetime changed is different from claiming a shift toward glycolysis or OXPHOS; the latter requires a well-characterized perturbation or independent validation.
- Include a mechanistically distinct control. Where an off-target biochemical signal is plausible— occupancy shifts from competing metabolites, pH or oxygen tension changes, rapid post-translational regulation—even in acute designs, pair the treatment of interest with a pharmacological control acting through a different, defined mechanism, and report pH, oxygenation, or phosphorylation-state alongside the lifetime data where relevant.
- Favor a paired, same-cell design. Comparing each cell or field before and after treatment exploits the “metabolic delta” approach and reduces non-flux-dependent variation.
- Validate enzymatic context independently when remodeling is plausible (beyond ~6–24 h, differentiation, chronic treatment). Quantitative proteomics is the most direct approach; transcriptomics, targeted immunofluorescence, or extracellular-flux (Seahorse/OCR) assays provide complementary functional validation.
- Acquire NAD(P)H and FAD together where feasible, to help separate redox-state changes from changes in total NAD(H) pool size.
- Report the modulation frequency used for phasor acquisition. Lower frequencies (e.g., 40 MHz vs. 80 MHz) compress the range of lifetimes represented on the universal semicircle, which can visually and quantitatively mask genuine rotation of the bound coordinate in prolonged experiments.
- Report the perturbation timescale explicitly, and state plainly which interpretive level— observational or mechanistic—the conclusions are pitched at.

## Conclusion

The two-state model of NADH fluorescence lifetime is a powerful and productive framework that has enabled a large body of metabolic imaging research. Its limitations are not a reason to abandon it, but to understand its domain of validity. In acute experimental settings, where the enzymatic landscape can be assumed stable, NADH lifetime reliably tracks shifts along the canonical metabolic axis. Across longer biological timescales, where enzyme expression can evolve, the signal integrates flux and enzymatic context in ways that require additional data to resolve. Importantly, this challenge is not restricted to long-term differentiation models. Many common experimental designs^19,20,25^, including 48–72 h pharmacological treatments, already operate within a temporal regime where adaptive enzymatic remodeling cannot be excluded. Making the timescale assumption explicit is therefore not a concern reserved for long-term biological models. It is a practical consideration for the design and interpretation of routine metabolic imaging experiments. The amount of biochemical context that must be known in advance will depend on the experiment. In some cases, the expected metabolic response to a treatment can be anticipated a priori from its known mechanism of action, allowing appropriate controls to be incorporated into the experimental design from the outset. In others, particularly for agents acting several steps removed from metabolism itself, this information becomes available only *a posteriori*, once independent proteomic, transcriptomic, or other data have been collected alongside the lifetime measurements, as in the longitudinal differentiation study discussed above.

As NADH FLIM is increasingly applied to metabolic reprogramming in differentiation, disease progression, and therapeutic response, matching the level of interpretation to the experimental timescale becomes a central requirement. The readout is not less valuable for being complex; rather, its value is strengthened when rapid, label-free imaging is combined with explicit consideration of the biochemical context that shapes the signal. Recent advances in sparse-photon FLIM reconstruction further show that acquisition speed and statistical rigor need not be traded off^29^. Within this framework, NADH FLIM can remain experimentally accessible while supporting more precise and defensible biological conclusions across timescales.

## Materials and methods

### Phasor simulation of proteome-driven trajectory rotation

NAD(P)H fluorescence was modelled on the phasor plot (modulation frequency 80 MHz; single-exponential mapping *g* = 1/(1 + (*ωτ*)²), *s* = *ωτ*/(1 + (*ωτ*)²)). Two cellular states, T0 and T1, were each described as a mixture of free NAD(P)H (*τ* = 0.4 ns) and ten generic enzyme-bound species with fixed bound lifetimes spaced from 1.25 to 4.50 ns (the upper bound approximating NADPH-bound states;^1,5,10,27^). Each species carried a molar amount *n*_*i*; the measured phasor point was the intensity-weighted mean of the component phasors, with intensity weight *n*_*i*·Φ_*i* and relative quantum yield Φ_*i* taken proportional to *τ*_*i* (constant radiative rate^14^). The molar free fraction was held equal in T0 and T1 (0.75), so that the phasor shift arose solely from the change in enzyme composition; T1 molar amounts differed from T0 by a fixed, interleaved pattern (up- and down-regulated enzymes distributed across the lifetime axis, fold changes 0.19–5.5, no species set to or from zero).

The classical two-component axis was defined as the line from the free-NADH phasor to the T0 bound centroid. The T0 → T1 displacement of the measured point was decomposed into a component parallel to this axis (*d*∥, the quantity a single-axis analysis attributes to a bound-fraction/redox change) and a perpendicular component (*d*⊥, the rotation). The trajectory rotation angle *θ* was measured at the common free-NADH vertex, between the free → T0 and free → T1 rays. The displacement between T0 and T1 centroids, Δ, and the fractional deviation *f* = |*d*⊥|/|Δ| were computed directly from these quantities.

Per-condition “experimental” point clouds were generated over 250 pixels: each pixel was assigned a molar free fraction drawn from *N* (0.75, 0.12) (truncated to [0.35, 0.96]; spatial heterogeneity of the bound fraction) and its phasor was computed from a periodic TCSPC decay (256 time channels over one modulation period) scaled to 400 expected photons and Poisson-sampled, giving 10⁵ photons per condition. The uncertainty on *θ* was obtained by propagating the centroid covariance (pixel covariance divided by the pixel count), projected perpendicular to the free → centroid ray. Simulations were implemented in Python 3 (NumPy, Matplotlib), fully deterministic given the random seed (20260827).

## Supplementary information

Transparent Peer Review File

Python Scripts Software

## Data Availability

No datasets were generated during the current study. The parameters used in the phasor simulation presented in Fig. 3 are described in full in the “Methods” section.

## Code availability

The custom Python code used to generate the phasor simulation presented in Fig. 3 is provided as Supplementary Data with this submission.
