# Supplementary material for: Interpreting NADH fluorescence lifetime across timescales
Source: Commun Biol. 2026 Nov 12;9:1518. doi: 10.1038/s42003-026-11344-z (PMC13759530; doi:10.1038/s42003-026-11344-z)
Supplement: Supplementary file 1 [file 42003_2026_11344_MOESM1_ESM.pdf]

nature portfolio

Peer Review File

Interpreting NADH fluorescence lifetime across timescales

Corresponding Author: Professor Francesco Cardarelli

This file contains all reviewer reports in order by version, followed by all author rebuttals in order by version.

Version 0:

Reviewer comments:

Reviewer #1

(Remarks to the Author)
Summary and overall assessment
This Perspective addresses a genuine and often overlooked problem in metabolic imaging: the conditions under which the free-to-bound NADH lifetime ratio can be read as a proxy for metabolic flux. The three-pillar framework (biochemical, spatial, and temporal assumptions) is a clear and useful pedagogical device, and the central argument is sound. Acute perturbations act on a fixed enzymatic landscape and can be interpreted within the two-state model, whereas processes that remodel the metabolic proteome (differentiation, prolonged pharmacological treatment) displace the bound-NADH endpoint on the phasor plot and reroute the metabolic trajectory, so that a lifetime shift conflates flux with enzymatic context. The longitudinal neuronal differentiation example is a persuasive proof of concept, and the practical guidelines are reasonable.
The conceptual framework is essentially correct, and I recommend publication after revision. However, the picture the manuscript draws is incomplete in three respects, and addressing them would substantially increase the rigor and the impact of the piece. My concerns are set out as three major comments, followed by a few minor points.

Major comments
1. The bound-NADH endpoint is modulated by small metabolites, not only by the enzymatic proteome
The manuscript attributes displacement of the bound-NADH coordinate almost entirely to remodeling of the enzymatic proteome, that is, to changes in the identity and abundance of NADH-binding oxidoreductases. This is only part of the story. The fluorescence lifetime of enzyme-bound NADH is determined by the conformation the coenzyme adopts within the binding pocket, and both that conformation and the fraction of NADH that is bound are modulated by small metabolites that participate in the catalytic cycle. Substrates, products, and inhibitors that form binary or ternary complexes shift the enzyme-NADH binding equilibrium and alter the bound-state lifetime with no change whatsoever in enzyme expression. Classic examples include the abortive ternary complexes formed by oxalate or oxamate with lactate dehydrogenase, and the complexes of malate or oxaloacetate with malate dehydrogenase.
This matters for the argument in two ways. First, these metabolite pools can change within seconds to minutes and have a genuine metabolic origin, so ligand-driven modulation of the bound endpoint is a distinct third axis of variation alongside metabolic flux and proteome remodeling. Second, and more importantly for the manuscript's logic, this modulation can operate within the very "acute" regime that the authors present as protected by biological inertia. The clean dichotomy (acute equals stable landscape equals interpretable; long-term equals remodeled proteome equals confounded) therefore understates the problem: the bound coordinate is not strictly fixed even on short timescales. I would encourage the authors to acknowledge this explicitly. It does not weaken their thesis; if anything it reinforces their broader point that the bound endpoint is dynamic and context dependent, while widening the scope of the caveat beyond proteome remodeling alone.

2. The metabolic meaning of the free-bound ratio has been validated at the whole-cell level, not at the subcellular scale
The metabolic interpretation of the free-to-bound ratio, and the anchoring of the bound coordinate by mitochondrial Complex I, has been established essentially at the level of the integrated whole-cell or whole-field signal. The manuscript relies heavily on the spatial pillar (mitochondrial signal dominance) to justify why the readout is bioenergetically meaningful, yet the metabolic meaning of the free-bound lifetime within distinct subcellular compartments (the mitochondrial matrix, the cytosol, the nucleus) has not, to my knowledge, been validated with comparable rigor. These compartments differ in NAD(H) pool size, redox poise, and enzyme complement, so the mapping between lifetime and metabolic state need not transfer across them.
This is increasingly relevant because the technique is being pushed toward subcellular and compartment-resolved claims. I suggest the authors state plainly that the biochemical mapping is validated at the ensemble level, and that its compartment-resolved interpretation remains an open question that will require dedicated study, for example by correlating compartment-

targeted optical measurements with independent biochemical readouts. Flagging this limitation would be consistent with the careful, boundary-setting spirit of the rest of the manuscript.

3. The change in bound lifetime over time is treated only as a limitation; it is also an opportunity, and the manuscript offers no spectroscopic route to address it
The manuscript diagnoses the central problem clearly: over long timescales the bound endpoint moves and the free-bound trajectory rotates onto a new axis. The only remedy it offers, however, is to acquire orthogonal proteomic or transcriptomic data. That is a valid recommendation, but it effectively concedes that the optical measurement alone cannot cope, and it leaves the most interesting possibility unexplored. The rotation of the free-bound trajectory is not merely a confound to be corrected away. The angle of the trajectory, and its evolution over time, itself carries biological information about which enzymatic species dominate the bound pool. To my knowledge, this angular dimension has not been the subject of systematic, repeated, longitudinal study.
I would encourage the authors to reframe the drift of the bound lifetime over time as an opportunity as well as a limitation, and to at least sketch how it might be approached from within the spectroscopic measurement itself, rather than deferring entirely to omics. The natural way to develop this properly would be a dedicated companion methods and spectroscopy paper: one that maps how the trajectory angle relates to enzymatic composition, and that tests whether the angular drift observed during differentiation or prolonged treatment is systematic and therefore interpretable in its own right. Even a short conceptual paragraph pointing in this direction would materially strengthen the present Perspective, which currently ends on a somewhat resigned note (the signal “requires additional data to resolve”) without indicating any optical or spectroscopic path toward that resolution.

Minor comments
Consistency of “invariant.” The temporal assumption is described in places as requiring a “strictly invariant” binding landscape and elsewhere as “predominantly invariant.” In light of major comment 1 (ligand-driven modulation on short timescales), the softer “predominantly invariant” phrasing is more defensible and should be used consistently.
Figure 2 legend. The legend attributes rotation of the metabolic trajectory to transcriptional and translational remodeling only. It would be more complete to note that ligand-induced changes in the binding equilibrium can also reorient the trajectory without any change in enzyme expression.

NADPH and pool-size confounds. These are introduced together in a dense passage. Since both are independent of the flux-versus-context distinction that is the core of the paper, a sentence clarifying that they are additional, separable confounds would help the reader keep the argument’s structure clear.

My recommendation is minor to moderate revision. The core argument is correct and the manuscript is a worthwhile contribution. Incorporating the three points above (in particular, acknowledging metabolite-driven modulation of the bound endpoint, flagging the whole-cell versus subcompartment validation gap, and offering a spectroscopic outlook on the trajectory-angle problem) would move the piece from a well-reasoned caution to a genuinely forward-looking Perspective.

Reviewer #2

(Remarks to the Author)
The manuscript by Ferri et al. discusses the limitations of metabolic-state characterization using FLIM. It is a concise and well-written article that could be particularly useful for junior researchers entering the field. However, the manuscript does not present any experimental data beyond the two schematic figures, which is somewhat unusual for a research article. This reviewer found Figures 1 and 2 highly informative and enjoyed reading and learning from the manuscript. Nevertheless, the authors may wish to consider expanding the manuscript into a review article. In addition to Figures 1 and 2, the authors could incorporate results from their own previous publications or from studies by other groups to provide experimental support for key arguments, particularly the temporal assumption discussed throughout the manuscript. Alternatively, the current format and scope would also be well suited for a book chapter.
This reviewer leaves the final decision regarding the suitability of this article format to the editor. If the editor considers this type of manuscript appropriate for publication in Communications Biology, I would support its publication.

Minor concerns:
1. On page 3, the authors state: “In the phasor framework, this weighted average determines the actual position of the ‘intracellular bound NADH’ pool (Figure 1b).” This reviewer suggests indicating the “weighted average” and/or the “intracellular bound NADH pool” directly in Figure 1b to make this concept easier for readers to identify.
2. Because the temporal assumption is a central argument of the manuscript, appropriate references should be provided whenever specific timescales are mentioned. For example, citations should be included to support the “48-72 hour pharmacological treatments” discussed on page 9 and the “6-24 hours” timescale mentioned on page 5.

Reviewer #3

(Remarks to the Author)
This Perspective discusses an important and timely issue in the interpretation of NADH fluorescence lifetime imaging: the extent to which lifetime shifts can be assigned to metabolic activity versus changes in the NADH-binding enzymatic landscape. The manuscript is clearly motivated and highlights a real limitation in the common free/bound NADH interpretation framework, especially in long-term differentiation, chronic treatment, and cellular adaptation experiments.

However, the current manuscript would benefit from a more balanced treatment of the field, clearer definitions of the proposed timescale boundaries, and more practical guidance for experimental design and reporting.
1. The manuscript suggests that acute perturbations can be interpreted with confidence because the enzymatic landscape is stable, whereas long-term treatments violate this assumption. While this is broadly reasonable, acute experiments can also be affected by pH, oxygenation, NADPH contribution, mitochondrial membrane potential, and rapid post-translational regulation. The authors should moderate the language and clarify that short timescales reduce, but do not eliminate, interpretive confounders.
2. The proposed temporal threshold requires stronger justification. The manuscript implies that adaptive changes in enzyme abundance typically require 6–24 hours or more, and that treatments of 24 hours or longer enter a regime where proteomic remodeling must be considered. This boundary is useful as a conceptual guideline, but it is likely cell-type-, stimulus-, and pathway-dependent. The authors should avoid presenting this threshold as a general rule and instead frame it as an approximate risk boundary. A simple table summarizing “minutes,” “hours,” “24–72 hours,” and “differentiation/developmental timescales” with recommended controls would make the Perspective more useful.
3. The manuscript criticizes overly simple assignments of free NADH to glycolysis and bound NADH to oxidative phosphorylation, but at several points it still relies on this classical mapping in fairly strong terms. The authors should more explicitly discuss cytosolic enzyme-bound NADH, lactate dehydrogenase and other oxidoreductases, mitochondrial matrix heterogeneity, NADH/NADPH overlap.
4. The literature coverage is somewhat narrow and should be more balanced. The manuscript relies heavily on the authors’ conceptual framing and cites a recent longitudinal neuronal differentiation study as the key direct evidence. The authors should discuss recent methodological advances that directly address practical limitations of NADH FLIM in low-photon, sparse, or large-scale biological imaging settings (Commun Biol 7, 1359 (2024); Theranostics, 2020, 10(4): 1849).
5. The discussion of proteomic remodeling is persuasive but would benefit from a more quantitative illustration. Since the main claim is that changes in the NADH-binding enzyme composition can move the bound-NADH phasor endpoint and mimic or distort metabolic interpretation, the manuscript should include a simple simulated phasor example or schematic calculation showing how reweighting several enzyme-bound lifetime components could produce an apparent shift along or across the classical metabolic trajectory. This would strengthen the biophysical basis of the Perspective.
6. The final section advises researchers to define the experimental regime, define the interpretation level, and characterize enzymatic context when remodeling is expected. These are appropriate principles, but the article would be more impactful if the authors provided a concrete checklist. For example, recommended controls could include acute inhibitor controls, paired pre/post measurements, NAD(P)H plus FAD imaging, NAD+/NADH or NADP+/NADPH biochemical assays, mitochondrial segmentation, oxygen or pH controls, Seahorse or respirometry validation, proteomics or targeted immunostaining for long-term studies, and explicit reporting of excitation wavelength, emission filters, fitting or phasor parameters, and photon statistics.
7. The figures are conceptually clear but risk oversimplifying the underlying biology. Figures 1 and 2 present the phasor trajectory and bound-NADH endpoint in an intuitive way, but the graphical arrows may imply a level of determinism that the text argues against. The authors should revise the figure legends to state explicitly that these trajectories are conceptual and not universal. The figure labels and annotations should also be enlarged and made more readable, especially for journal-scale reproduction.

Version 1:

Reviewer comments:

Reviewer #1

(Remarks to the Author)
The perspective has improved in the revised version. Still, there are a few minor comments the authors need to address. Please read the documents carefully to proofread the text and use the agreed format for em dash (—), as added in the text, because it is sometimes used as em dash (—, long) and sometimes as dash (–, short).

Reviewer #3

(Remarks to the Author)
I find that the authors have put considerable effort into addressing the reports of the referees. As a result the paper is very much improved and I have no problem in recommending it for publication.

Open Access This Peer Review File is licensed under a Creative Commons Attribution 4.0 International License, which permits use, sharing, adaptation, distribution and reproduction in any medium or format, as long as you give appropriate credit to the original author(s) and the source, provide a link to the Creative Commons license, and indicate if changes were made.
In cases where reviewers are anonymous, credit should be given to 'Anonymous Referee' and the source.
The images or other third party material in this Peer Review File are included in the article's Creative Commons license, unless indicated otherwise in a credit line to the material. If material is not included in the article's Creative Commons license and your intended use is not permitted by statutory regulation or exceeds the permitted use, you will need to obtain permission directly from the copyright holder.
To view a copy of this license, visit https://creativecommons.org/licenses/by/4.0/

Reviewers' comments:

Reviewer #1 (Remarks to the Author):

Summary and overall assessment
This Perspective addresses a genuine and often overlooked problem in metabolic imaging: the conditions under which the free-to-bound NADH lifetime ratio can be read as a proxy for metabolic flux. The three-pillar framework (biochemical, spatial, and temporal assumptions) is a clear and useful pedagogical device, and the central argument is sound. Acute perturbations act on a fixed enzymatic landscape and can be interpreted within the two-state model, whereas processes that remodel the metabolic proteome (differentiation, prolonged pharmacological treatment) displace the bound-NADH endpoint on the phasor plot and reroute the metabolic trajectory, so that a lifetime shift conflates flux with enzymatic context. The longitudinal neuronal differentiation example is a persuasive proof of concept, and the practical guidelines are reasonable.
The conceptual framework is essentially correct, and I recommend publication after revision. However, the picture the manuscript draws is incomplete in three respects, and addressing them would substantially increase the rigor and the impact of the piece. My concerns are set out as three major comments, followed by a few minor points.

We thank the Reviewer for the careful and constructive assessment, and for recognizing the value of the three-pillar framework and the central argument of the Perspective. We have used the review to broaden the framework beyond proteome remodeling alone, clarify its spatial domain of validation, and develop a more forward-looking spectroscopic outlook.

Major comments
1. The bound-NADH endpoint is modulated by small metabolites, not only by the enzymatic proteome
The manuscript attributes displacement of the bound-NADH coordinate almost entirely to remodeling of the enzymatic proteome, that is, to changes in the identity and abundance of NADH-binding oxidoreductases. This is only part of the story. The fluorescence lifetime of enzyme-bound NADH is determined by the conformation the coenzyme adopts within the binding pocket, and both that conformation and the fraction of NADH that is bound are modulated by small metabolites that participate in the catalytic cycle. Substrates, products, and inhibitors that form binary or ternary complexes shift the enzyme-NADH binding equilibrium and alter the bound-state lifetime with no change whatsoever in enzyme expression. Classic examples include the abortive ternary complexes formed by oxalate or oxamate with lactate dehydrogenase, and the complexes of malate or oxaloacetate with malate dehydrogenase.
This matters for the argument in two ways. First, these metabolite pools can change within seconds to minutes and have a genuine metabolic origin, so ligand-driven modulation of the bound endpoint is a distinct third axis of variation alongside metabolic flux and

proteome remodeling. Second, and more importantly for the manuscript's logic, this modulation can operate within the very "acute" regime that the authors present as protected by biological inertia. The clean dichotomy (acute equals stable landscape equals interpretable; long-term equals remodeled proteome equals confounded) therefore understates the problem: the bound coordinate is not strictly fixed even on short timescales. I would encourage the authors to acknowledge this explicitly. It does not weaken their thesis; if anything it reinforces their broader point that the bound endpoint is dynamic and context dependent, while widening the scope of the caveat beyond proteome remodeling alone.

Response: We thank the Reviewer for this interesting comment which actually stimulated us to improve the manuscript. A new subsection, 'Caveats even within the acute regime,' now immediately follows the acute-regime discussion and explicitly identifies ligand- or metabolite-driven occupancy shifts as a distinct axis of variation that operates on a timescale of seconds to minutes and is not protected by the biological inertia that shields enzyme identity and abundance. We extended this treatment to other fast-acting, off-target biochemical signals - pH, oxygen tension, and rapid post-translational regulation (e.g., phosphorylation) - and illustrate all of them in a new Figure 2b. We also note, in the same section, that the magnitude of any such occupancy-driven displacement scales with how much the affected enzyme contributes to the aggregate bound signal, connecting this caveat explicitly to the spatial (mitochondrial-dominance) pillar.

2. The metabolic meaning of the free-bound ratio has been validated at the whole-cell level, not at the subcellular scale
The metabolic interpretation of the free-to-bound ratio, and the anchoring of the bound coordinate by mitochondrial Complex I, has been established essentially at the level of the integrated whole-cell or whole-field signal. The manuscript relies heavily on the spatial pillar (mitochondrial signal dominance) to justify why the readout is bioenergetically meaningful, yet the metabolic meaning of the free-bound lifetime within distinct subcellular compartments (the mitochondrial matrix, the cytosol, the nucleus) has not, to my knowledge, been validated with comparable rigor. These compartments differ in NAD(H) pool size, redox poise, and enzyme complement, so the mapping between lifetime and metabolic state need not transfer across them.
This is increasingly relevant because the technique is being pushed toward subcellular and compartment-resolved claims. I suggest the authors state plainly that the biochemical mapping is validated at the ensemble level, and that its compartment-resolved interpretation remains an open question that will require dedicated study, for example by correlating compartment-targeted optical measurements with independent biochemical readouts. Flagging this limitation would be consistent with the careful, boundary-setting spirit of the rest of the manuscript.

Response: We agree with the Reviewer. We have added an explicit statement to this effect in the spatial-assumption section: the mitochondrial-dominance argument is typically invoked and validated at the scale of the whole cell or imaging field, often pooling many cells within a single frame, and we now state plainly that whether the same weighting holds within individual cells, or varies systematically across subcellular compartments, has not been addressed systematically.

3. The change in bound lifetime over time is treated only as a limitation; it is also an opportunity, and the manuscript offers no spectroscopic route to address it

The manuscript diagnoses the central problem clearly: over long timescales the bound endpoint moves and the free-bound trajectory rotates onto a new axis. The only remedy it offers, however, is to acquire orthogonal proteomic or transcriptomic data. That is a valid recommendation, but it effectively concedes that the optical measurement alone cannot cope, and it leaves the most interesting possibility unexplored. The rotation of the free-bound trajectory is not merely a confound to be corrected away. The angle of the trajectory, and its evolution over time, itself carries biological information about which enzymatic species dominate the bound pool. To my knowledge, this angular dimension has not been the subject of systematic, repeated, longitudinal study.

I would encourage the authors to reframe the drift of the bound lifetime over time as an opportunity as well as a limitation, and to at least sketch how it might be approached from within the spectroscopic measurement itself, rather than deferring entirely to omics. The natural way to develop this properly would be a dedicated companion methods and spectroscopy paper: one that maps how the trajectory angle relates to enzymatic composition, and that tests whether the angular drift observed during differentiation or prolonged treatment is systematic and therefore interpretable in its own right. Even a short conceptual paragraph pointing in this direction would materially strengthen the present Perspective, which currently ends on a somewhat resigned note (the signal “requires additional data to resolve”) without indicating any optical or spectroscopic path toward that resolution.

Response: We thank the Reviewer for this suggestion, which we have adopted as one of the central additions of the revision. We now argue, in a new section titled ‘Beyond the breakdown: quantifying drift without resolving its cause,’ that the displacement of the bound-NADH coordinate can be decomposed geometrically with respect to the original free-to-bound trajectory into a component along that axis and a component perpendicular to it, and that the perpendicular component, together with a rotation angle θ and a fractional deviation f, can be quantified independently of the specific combination of enzyme changes that produced it. We validated this quantitative claim with a phasor simulation of a ten-enzyme reference

system (new Figure 3, with a corresponding Methods section), showing that the resulting T0 and T1 point clouds remain clearly separated on the phasor plot even under a realistic photon budget. We agree that a fully dedicated companion methods paper, explicitly correlating trajectory angle with enzymatic composition across real longitudinal datasets, is the natural next step, and have flagged this as a direction for future work; the present simulation is intended as a first, self-contained proof of concept that the angular dimension is quantifiable in principle.

Minor comments
Consistency of “invariant.” The temporal assumption is described in places as requiring a “strictly invariant” binding landscape and elsewhere as “predominantly invariant.” In light of major comment 1 (ligand-driven modulation on short timescales), the softer “predominantly invariant” phrasing is more defensible and should be used consistently.

Response: Corrected. We now use ‘predominantly invariant’ consistently throughout, and we explicitly split the temporal assumption into its two components - enzyme identity/abundance and binding configuration - to make clear which is protected by biological inertia and which is not.

Figure 2 legend. The legend attributes rotation of the metabolic trajectory to transcriptional and translational remodeling only. It would be more complete to note that ligand-induced changes in the binding equilibrium can also reorient the trajectory without any change in enzyme expression.

Response: Thank you for this interesting comment, which actually stimulated us to thoroughly modify the manuscript. Rather than a caption note on the chronic-regime figure, ligand-driven occupancy shifts are now treated as a distinct mechanism in their own right: Figure 2b explicitly depicts an 'occupancy modulator' alongside the other fast, off-target biochemical signals discussed in the new 'Caveats even within the acute regime' section, making clear that trajectory rotation can arise from this mechanism independently of any proteomic remodeling.

NADPH and pool-size confounds. These are introduced together in a dense passage. Since both are independent of the flux-versus-context distinction that is the core of the paper, a sentence clarifying that they are additional, separable confounds would help the reader keep the argument’s structure clear.

Response: We thank the Reviewer for highlighting this possible source of confusion. To address it, we have added a sentence clarifying that both NADPH spectral overlap and total NAD(H) pool-size variations are compositional confounds that are independent of the flux-versus-context distinction central to the Perspective, and do not require any change in the enzymatic binding landscape to shift the measured lifetime.

My recommendation is minor to moderate revision. The core argument is correct and the manuscript is a worthwhile contribution. Incorporating the three points above (in particular, acknowledging metabolite-driven modulation of the bound endpoint, flagging the whole-cell versus subcompartment validation gap, and offering a spectroscopic outlook on the trajectory-angle problem) would move the piece from a well-reasoned caution to a genuinely forward-looking Perspective.

Reviewer #2 (Remarks to the Author):

The manuscript by Ferri et al. discusses the limitations of metabolic-state characterization using FLIM. It is a concise and well-written article that could be particularly useful for junior researchers entering the field. However, the manuscript does not present any experimental data beyond the two schematic figures, which is somewhat unusual for a research article. This reviewer found Figures 1 and 2 highly informative and enjoyed reading and learning from the manuscript. Nevertheless, the authors may wish to consider expanding the manuscript into a review article. In addition to Figures 1 and 2, the authors could incorporate results from their own previous publications or from studies by other groups to provide experimental support for key arguments, particularly the temporal assumption discussed throughout the manuscript. Alternatively, the current format and scope would also be well suited for a book chapter.
This reviewer leaves the final decision regarding the suitability of this article format to the editor. If the editor considers this type of manuscript appropriate for publication in Communications Biology, I would support its publication.

We thank the Reviewer for the positive assessment of the manuscript and figures, and for supporting publication if the Perspective format is considered appropriate by the Editor. The manuscript was submitted and evaluated as a Perspective, whose purpose is to advance a focused conceptual argument and provide interpretive guidance rather than to report a new experimental dataset. We have therefore retained this format, in line with the Editor's invitation to submit a revised Perspective. At the same time, we have strengthened its evidential and practical basis by broadening the literature coverage, adding a timescale-and-controls summary table, and expanding the discussion of the longitudinal evidence supporting the temporal argument.

Minor concerns:
1. On page 3, the authors state: “In the phasor framework, this weighted average determines the actual position of the ‘intracellular bound NADH’ pool (Figure 1b).” This reviewer suggests indicating the “weighted average” and/or the “intracellular bound NADH pool” directly in Figure 1b to make this concept easier for readers to identify.

Response: We agree and have added a 'weighted average' label directly on the relevant point in Figure 1b, alongside its existing biological label ('mitochondrial oxidative metabolism'), and revised the corresponding caption sentence to state explicitly that the bound reference point is labeled 'weighted average' in the panel, so that readers can connect the concept in the text with its visual location.

2. Because the temporal assumption is a central argument of the manuscript, appropriate references should be provided whenever specific timescales are mentioned. For example, citations should be included to support the “48-72 hour pharmacological treatments” discussed on page 9 and the “6-24 hours” timescale mentioned on page 5.

Response: Thank you for this suggestion. We have added supporting citations at both timescales: Irrcher et al. (2021, Front Mol Biosci) for the 6–24 hour threshold, and Walsh et al. (2021, Cancer Res) and Fong et al. (2021, SLAS Discovery) for the 48–72 hour pharmacological-treatment timescale.

Reviewer #3 (Remarks to the Author):

This Perspective discusses an important and timely issue in the interpretation of NADH fluorescence lifetime imaging: the extent to which lifetime shifts can be assigned to metabolic activity versus changes in the NADH-binding enzymatic landscape. The manuscript is clearly motivated and highlights a real limitation in the common free/bound NADH interpretation framework, especially in long-term differentiation, chronic treatment, and cellular adaptation experiments. However, the current manuscript would benefit from a more balanced treatment of the field, clearer definitions of the proposed timescale boundaries, and more practical guidance for experimental design and reporting.

We thank the Reviewer for recognizing the importance and timeliness of the Perspective. In response to these constructive comments, we have revised the manuscript throughout to provide a more balanced and context-dependent account of NADH FLIM interpretation. Specifically, we have moderated the distinction between acute and long-term experiments, reframed the proposed timescale boundaries as approximate and context-dependent risk boundaries, expanded the discussion of molecular and optical determinants of fluorescence lifetime, broadened the literature coverage, and added both a summary table and a boxed practical checklist for experimental design and reporting. Together, these revisions clarify the scope and limitations of the proposed framework and make its guidance more directly applicable across different experimental settings.

1. The manuscript suggests that acute perturbations can be interpreted with confidence because the enzymatic landscape is stable, whereas long-term treatments violate this assumption. While this is broadly reasonable, acute experiments can also be affected by pH, oxygenation, NADPH contribution, mitochondrial membrane potential, and rapid post-translational regulation. The authors should moderate the language and clarify that short timescales reduce, but do not eliminate, interpretive confounders.

Response: We thank the Reviewer for this interesting comment. To address it, we have added a full subsection, 'Caveats even within the acute regime,' that explicitly moderates this language: it states that the acute regime is protected against confounding by proteomic drift specifically, not immune to every source of drift in the bound endpoint, and discusses pH, oxygen tension, rapid post-translational regulation, and ligand-driven occupancy shifts as fast, off-target biochemical signals that remain possible even in acute designs.

2. The proposed temporal threshold requires stronger justification. The manuscript implies that adaptive changes in enzyme abundance typically require 6–24 hours or more, and that treatments of 24 hours or longer enter a regime where proteomic remodeling must be

considered. This boundary is useful as a conceptual guideline, but it is likely cell-type-, stimulus-, and pathway-dependent. The authors should avoid presenting this threshold as a general rule and instead frame it as an approximate risk boundary. A simple table summarizing “minutes,” “hours,” “24–72 hours,” and “differentiation/developmental timescales” with recommended controls would make the Perspective more useful.

Response: We agree and have reframed the threshold throughout as an approximate indicator of risk rather than a sharp cutoff, softening the surrounding language accordingly. We have also added Table 1, which summarizes four experimental regimes (acute, short, intermediate ~6–24 h, long >24–72 h and beyond) alongside their main risk to the temporal assumption and recommended safeguards.

3. The manuscript criticizes overly simple assignments of free NADH to glycolysis and bound NADH to oxidative phosphorylation, but at several points it still relies on this classical mapping in fairly strong terms. The authors should more explicitly discuss cytosolic enzyme-bound NADH, lactate dehydrogenase and other oxidoreductases, mitochondrial matrix heterogeneity, NADH/NADPH overlap.

Response: We agree and have substantially expanded the biochemical-assumption discussion to address each of these points explicitly: the non-negligible cytosolic bound fraction (contributed chiefly by lactate dehydrogenase and other cytosolic oxidoreductases), heterogeneity of the mitochondrial matrix itself across individual mitochondria and subregions, and NADH/NADPH spectral overlap, including a discussion of when this overlap is, and is not, expected to be negligible.

4. The literature coverage is somewhat narrow and should be more balanced. The manuscript relies heavily on the authors’ conceptual framing and cites a recent longitudinal neuronal differentiation study as the key direct evidence. The authors should discuss recent methodological advances that directly address practical limitations of NADH FLIM in low-photon, sparse, or large-scale biological imaging settings (Commun Biol 7, 1359 (2024); Theranostics, 2020, 10(4): 1849).

Response: We have added the suggested citations (Shen et al., Commun Biol 7, 1359 (2024); Shen et al., Theranostics 10(4), 1849 (2020)) in the Conclusion and spatial-assumption sections respectively, discussing sparse-photon FLIM reconstruction and large-field optical platforms as practical routes to reconcile

acquisition speed with statistical rigor.

5. The discussion of proteomic remodeling is persuasive but would benefit from a more quantitative illustration. Since the main claim is that changes in the NADH-binding enzyme composition can move the bound-NADH phasor endpoint and mimic or distort metabolic interpretation, the manuscript should include a simple simulated phasor example or schematic calculation showing how reweighting several enzyme-bound lifetime components could produce an apparent shift along or across the classical metabolic trajectory. This would strengthen the biophysical basis of the Perspective.

Response: We agree. This is now addressed by the new phasor simulation described in response to Reviewer #1's third major comment above (new Figure 3 and accompanying Methods section). The simulation reweights a ten-enzyme reference system between two time points and shows explicitly how the resulting bound coordinate departs from the classical free-to-bound trajectory, providing a concrete, quantitative illustration of the mechanism the manuscript describes conceptually.

6. The final section advises researchers to define the experimental regime, define the interpretation level, and characterize enzymatic context when remodeling is expected. These are appropriate principles, but the article would be more impactful if the authors provided a concrete checklist. For example, recommended controls could include acute inhibitor controls, paired pre/post measurements, NAD(P)H plus FAD imaging, NAD+/NADH or NADP+/NADPH biochemical assays, mitochondrial segmentation, oxygen or pH controls, Seahorse or respirometry validation, proteomics or targeted immunostaining for long-term studies, and explicit reporting of excitation wavelength, emission filters, fitting or phasor parameters, and photon statistics.

Response: Thank you for this comment. We have added Box 1, a concrete, itemized checklist for experimental design and reporting: defining the experimental regime and level of interpretation, including a mechanistically distinct control where an off-target biochemical signal is plausible, favoring a paired same-cell design, validating enzymatic context independently when remodeling is plausible (with Seahorse/OCR assays noted as a complementary functional check rather than a substitute for proteomic validation), acquiring NAD(P)H and FAD together, reporting the modulation frequency used for phasor acquisition, and reporting the perturbation timescale and interpretive level explicitly.

7. The figures are conceptually clear but risk oversimplifying the underlying biology. Figures 1 and 2 present the phasor trajectory and bound-NADH endpoint in an intuitive way, but the graphical arrows may imply a level of determinism that the text argues against. The authors should revise the figure legends to state explicitly that these trajectories are conceptual and not universal. The figure labels and annotations should also be enlarged and made more readable, especially for journal-scale reproduction.

Response: Thank you for this comment. We have revised the legends of Figures 1 and 2 to state explicitly that the depicted trajectories and schematics are conceptual rather than universal. Figure 3 is not schematic in this sense, as it presents the output of an explicit phasor simulation rather than an illustrative trajectory, so we have not added the same qualifier there. Regarding figure labels, all annotations are set at 8 pt or larger on artboards sized to final print dimensions, consistent with the journal's minimum requirements for figure text.

Point-by-point response

We thank the Editor and the Reviewers for their time in reviewing the revised manuscript. Below we respond to each Reviewer's comments.

Reviewer #1 (Remarks to the Author):

The perspective has improved in the revised version. Still, there are a few minor comments the authors need to address. Please read the documents carefully to proofread the text and use the agreed format for em dash (—), as added in the text, because it is sometimes used as em dash (— , long) and sometimes as dash (–, short).

Response: We thank the Reviewer for catching this. We have reviewed the entire manuscript and standardized every parenthetical/clause-break dash to the em dash (—); number ranges (e.g., 1.25-4.5 ns, 6-24 hours) correctly retain the en dash and were left unchanged. The corrected passages are marked in the accompanying tracked-changes file.

Reviewer #3 (Remarks to the Author):

I find that the authors have put considerable effort into addressing the reports of the referees. As a result the paper is very much improved and I have no problem in recommending it for publication.

Response: We thank the Reviewer for this assessment and for their time throughout the review process.
